# CAD-Based Analysis and Experimental Validation of Registration Errors in Imageless Total Knee Arthroplasty

**DOI:** 10.3390/jcm15062335

**Published:** 2026-03-18

**Authors:** Muhammad Sohail, Salman Khalid, Muhammad Umar Elahi, Heung Soo Kim

**Affiliations:** 1 Department of Mechanical, Robotics and Energy Engineering, Dongguk University-Seoul, 30 Pildong-ro 1-gil, Jung-gu, Seoul 04620, Republic of Korea; 2 Department of Civil and Environmental Engineering, University of Michigan, Ann Arbor, MI 48109, USA

**Keywords:** knee arthroplasty, computational simulation, knee surgery, experimental validation, registration error, imageless navigator

## Abstract

**Background/Objectives**: Accurate implant positioning in total knee arthroplasty (TKA) depends on reliable intraoperative landmark registration. In imageless TKA, registration errors can alter cutting-plane orientation and compromise alignment. This study quantitatively evaluated how anatomical landmark registration errors affect cutting-plane orientation in imageless TKA. **Methods**: A CAD-based simulation with controlled experimental validation using 3D-printed bone models was performed to reproduce the imageless TKA workflow. Controlled errors were introduced into key femoral and tibial landmarks, and the resulting deviations were quantified. The primary evaluation metrics were angular deviations in varus/valgus, flexion/extension, and internal/external rotation. **Results**: Coronal and rotational alignment showed the greatest sensitivity to registration error. In the femur, anteroposterior epicondylar displacement had the strongest rotational influence, with sensitivity reaching about 0.5°/mm, whereas mediolateral displacement of the tibial anteroposterior landmarks showed the highest sensitivity at about 1.4°/mm. Similar trends were observed in both simulation and experimental validation cases. **Conclusions**: The findings indicate that small registration errors can produce clinically significant cutting-plane deviations in imageless TKA, particularly at the femoral transepicondylar and tibial anteroposterior landmarks, and may approach commonly accepted alignment thresholds.

## 1. Introduction

Due to its multi-axial movement and structural anatomy, the knee joint exhibits considerable mechanical complexity. Because of its structural complexity and high loading, the knee is prone to degenerative conditions, such as osteoarthritis, that results in pain and discomfort to the patient [1]. In severe stages of knee degeneration, total knee arthroplasty (TKA) is commonly recognized as the most effective intervention for pain relief and functional restoration [2]. TKA can be performed using image-guided techniques, such as ultrasound [3], or through imageless computer-assisted systems [4]. Computer-assisted TKA has gained increasing adoption because of its potential to improve alignment accuracy [5]. These systems typically utilize specialized software that registers key anatomical landmarks. Localization of these points is achieved through tracking technologies that employ infrared, laser, or electromagnetic signals, which are detected via cameras or sensors to capture the real-time spatial coordinates of the reference markers [6,7,8].

In imageless navigation, registered anatomical landmarks are used to establish virtual coordinate systems of the distal femur and proximal tibia, which serve as the reference frameworks for defining cutting-plane orientation and alignment during surgery [9]. The precision of these frameworks is critically influenced by the accuracy with which these anatomical landmarks are identified [10]. Error in landmark registration, whether due to intraoperative variability, poor visibility, or surgeon judgment, can lead to substantial rotational misalignment [11,12,13]. As the coordinate systems are constructed from these registered points, errors in landmark localization can propagate to the orientation of the cutting-plane and affect implant alignment. The femoral coordinate system (FCS) is commonly established using the femoral mechanical axis together with a femoral transverse axis [14]. In the literature, the femoral mechanical axis is typically defined as the vector joining the femoral head center (FHC) and the knee center (KC), whereas the femoral transverse axis has been defined using several anatomical or geometric references, each with practical limitations related to landmark visibility, reproducibility, and degenerative changes [11,12,15,16,17]. As with femoral landmark registration, tibial landmark registration directly affects the coordinate-system construction used for cutting-plane orientation and alignment calculations in imageless TKA. The tibial coordinate system (TCS) is generally constructed using the tibial mechanical axis, commonly defined by the tibial center (TC) and the center of the malleoli (CM), together with a second reference axis defined either by the anteroposterior (AP) axis or the lateromedial axis defined by the tibial medial and lateral plateaus [14,18]. Since cutting-plane orientation and alignment calculations are performed with respect to the FCS and TCS [9], inaccuracies in landmark localization may propagate directly to angular deviations in varus/valgus, flexion/extension, and internal/external rotation [19]. In this work, registration error refers to a point-localization error at a specific anatomical landmark, and is modeled as a controlled translational shift of that landmark in defined anatomical directions.

Previous studies have shown that femoral landmark registration in TKA is subject to considerable variability, even under controlled conditions, thereby indicating a substantial possibility of registration error during anatomical landmark identification. Particularly for landmarks used to define the femoral transverse axis in osteoarthritic knees, where degenerative changes can make them harder to identify reliably [11,12,17,20]. Experimental and cadaveric studies have further shown that misregistration of epicondylar landmarks can produce substantial errors in femoral axis definition and alignment deviations, in some cases exceeding the commonly accepted 3° threshold [13,21]. For the tibial landmark registration, studies in imageless navigated TKA have shown that registration errors at the proximal tibial center can produce projected errors of up to 1.3° in the coronal plane and 2.0° in the sagittal plane [22], while repeated registration of proximal tibial and malleolar landmarks has further confirmed observer-dependent tibial registration error [23]. However, systematic landmark-specific quantification of tibial error propagation remains limited. Although these findings show that landmark identification error can influence component alignment, most prior work has focused primarily on femoral references. A systematic quantitative assessment of how directional registration errors at both femoral and tibial landmarks propagate to varus/valgus, flexion/extension, and internal/external rotation remains limited. Moreover, studies combining controlled CAD-based analysis with experimental validation to investigate this error propagation are still lacking.

This study aims to conduct a quantitative evaluation of angular deviations caused by inaccuracies in anatomical landmark registration during TKA. A controlled simulation model of the distal femur and proximal tibia is developed, where key anatomical landmarks systematically incremented along defined anatomical directions. The resulting deviations in flexion/extension, varus/valgus, and internal/external rotation are analyzed. To validate the simulation findings, an experimental setup using optical navigation tools is employed. This study provides an understanding of which anatomical regions and directional shifts most significantly impact alignment, highlighting the directional sensitivity of both femoral and tibial coordinate systems. It was hypothesized that registration errors would affect the alignment parameters unequally, with superior/inferior registration errors at most landmarks producing minimal angular deviation, while internal/external rotation would be particularly sensitive to errors influencing the lateromedial transverse axis.

## 2. Materials and Methods

### 2.1. Overview of the Imageless Navigation Algorithm

The input to the algorithm consists of anatomical landmarks at the distal femur and proximal tibia. To construct the FCS, the algorithm employs a vector algebra-based method using key anatomical points at the distal femur, including the FHC, ME, and LE, and the KC. Let $r_{FHC}$, $r_{KC}$, $r_{LE}$ and $r_{ME}$ denote the 3D positions of these landmarks in the global frame. The mechanical axis of the femur, which defines the FHC **y-axis**, is given by Equations (1)–(5) [24,25]:

(1) $$y_{FHC}=\frac{r_{FHC}-r_{KC}}{∥r_{FCS}-r_{KC}∥}$$

The aTEA is defined by the line joining the LE and ME. Its directional vector is given by:

(2) $$t_{FHC}=\frac{r_{LE}-r_{ME}}{∥r_{LE}-r_{ME}∥}$$

To ensure the orthogonality, the component of $t_{FCS}$ perpendicular to the mechanical axis is obtained as:

(3) $$z_{FHC}=\frac{t_{FCS}-(t_{FCS}\cdot y_{FCS})y_{FCS}}{∥t_{FCS}-(t_{FCS}\cdot y_{FCS})y_{FCS}∥}$$

This projection step is necessary to define a mathematically consistent orthogonal coordinate system. Once $y_{FCS}$ and $z_{FCS}$ are defined, the cross product completes the FCS. The same procedure is applied at the proximal tibia, using the TC, CM, and AP axis to construct the TCS. Once FCS and TCS are defined, local coordinate systems (LCS) are established at the fixed and probe markers to enable real-time computation of bone orientation and cutting depth.

The varus/valgus and flexion/extension are evaluated in the coronal and sagittal planes, respectively. Therefore, the relevant vectors (femur or tibia mechanical axis) are first projected onto these anatomical planes. For an arbitrary vector v, the projections onto the femoral coronal (yz-plane) and sagittal (xy-plane) are defined as:

(4) $$v^{FCS\Pi_{yz},proj}=v-t_{FCS}-\left(v.x_{FCS}\right)x_{FCS}$$

(5) $$v^{FCS\Pi_{xy},proj}=v-t_{FCS}-\left(v.z_{FCS}\right)z_{FCS}$$

**Figure 1:**
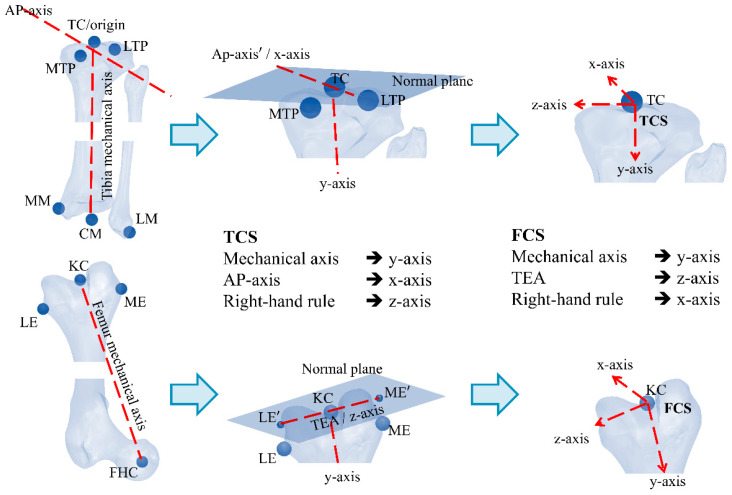
A generalized method to assign femoral and tibial coordinate systems.

Figure 1 illustrates the generalized methodology for assigning TCS and FCS. The setting of the resection plane consists of the calculation of two angles and one distance (cutting depth). This involves calculation of varus/valgus (rotation in coronal plane about anteroposterior axis), and the flexion angles (rotation in sagittal plane about lateromedial axis). The cutting angles can be calculated as follows [9,26,27].

(6) $$varus=90{}^{\circ}-cos^{-1}\left(\frac{z_{FCS}\cdot y_{FCS\prime }^{FCS_{\Pi yz}},proj.}{∥z_{FCS}∥\cdot ∥y_{FCS\prime }^{FCS_{\Pi yz}},proj.∥}\right)$$

(7) $$flexion=cos^{-1}\left(\frac{x_{FCS}\cdot y_{FCS^{\prime }}^{FCS_{\Pi xy}},proj.}{∥x_{FCS}∥\cdot ∥y_{FCS^{\prime }}^{FCS_{\Pi xy}},proj.∥}\right)-90{}^{\circ}$$

where $y_{FCS^{\prime }}$ and $z_{FCS}$ are the y and z axes of the femoral coordinate system with registration error and femoral coordinate systems without any registration error, $y_{FCS^{\prime }}^{FCS_{\Pi yz}},proj.$ and $y_{FCS^{\prime }}^{FCS_{\Pi xy}},proj.$ are the projections of the *y*-axis on femoral yz and xy planes, respectively. The algorithm employed in this study is based on the comprehensive vector-based framework described in Algorithm 1, which was experimentally evaluated using a sawbone-based setup [9].
**Algorithm 1** Method to calculate registration errors.**Function Calculate registration error**  *// Following coordinate system is defined using accurate anatomical points*  **FCS_standard_ := Define local bone coordinate** (KC, FHC, LE, ME)  *// Adding error in anatomical landmarks (one at a time)*  **For** each KC′ in Perturbed_KC_List do    **FCS_Perturbed_ := Define local bone coordinate** (KC′, FHC, LE, ME)    Deviations **= Calculate cutting angles** (FCS_standard_, FCS_Perturbed_)    Save Deviations *        // Store Varus/Flexion angle results*  **end For** **end Function** **Function Define local bone coordinate** (origin, point 1, point 2, point 3)  **y-axis** ← point 1—origin  Plane := **y axis**, origin *        // Define a plane normal to **y-axis** and pass*                    **//**
*through origin*  point2′ = Project point2 on Plane *   // Due to this projection the superior/inferior*
  point3′ = Project point3 on Plane *   // translation effect is minimal*  **z-axis** ← point 2′–point 3′  **x-axis** ← Cross (y axis, z axis)  **return** [**x-axis, y-axis, z-axis**] **end Function** **Function Calculate cutting angles** (FCS_standard_, FCS_Perturbed_)  $Varus\leftarrow angle\left(y_{{FCS}_{perturbed}}^{{FCS}_{\begin{array}{c} {standard}_{\Pi_{yz}} \end{array}}},proj.\&z_{{FCS}_{standard}}\right)$ *        // Varus is calculated in*                              ***//***
*coronal plane*  $Flexion\leftarrow angle\left(y_{{FCS}_{perturbed}}^{{FCS}_{\begin{array}{c} {standard}_{\Pi_{xy}} \end{array}}},proj.\&x_{{FCS}_{standard}}\right)$ *        // Flexion is calculated in*                              ***//***
*sagittal plane*  **return** [Varus; Flexion] **end Function**

### 2.2. CAD Modeling and Landmark Registration

**Figure 2:**
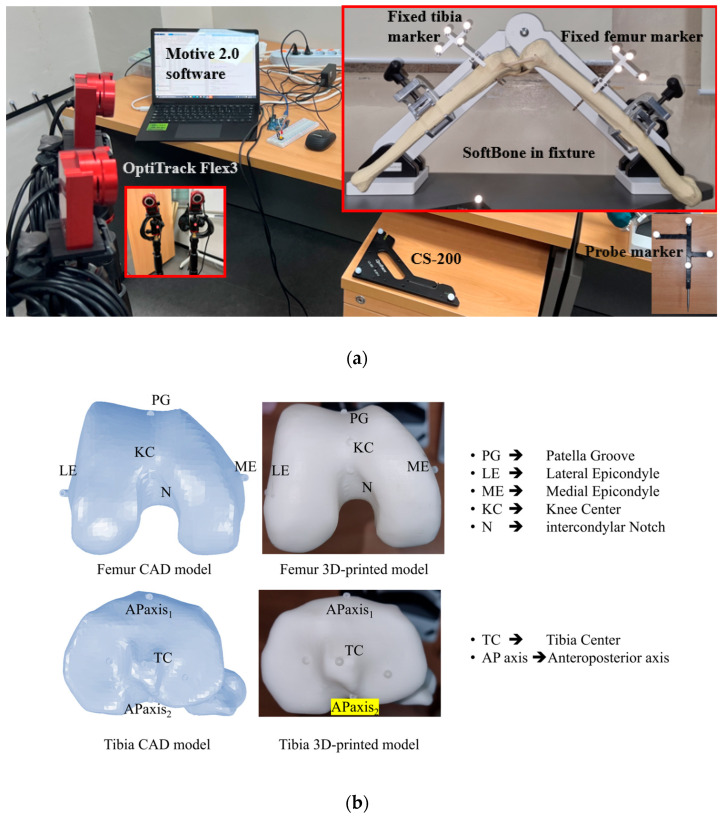
(**a**) 3D printed bone models, (**b**) CT scanned CAD bone.

One CT-scan-based 3D model of a human lower limb was used to develop the CAD framework [28], and one corresponding set of 3D-printed femoral and tibial specimens was produced for experimental validation. A single model set was used in this proof-of-concept study to isolate landmark-specific error propagation under controlled geometric conditions and to enable direct comparison between CAD predictions and experimental measurements without inter-subject anatomical variability. Anatomical landmarks were manually identified on both the femur and tibia using standard anatomical references based on well-defined criteria in the literature [29]. On the femur, the FHC, KC, LE, and ME were marked to define the FCS. On the tibia, the TC, lateral malleoli (LM), medial malleoli (MM), and AP-axis were identified to construct the TCS. Following the landmark registration, the marked 3D models of the femur and tibia were 3D printed to enable physical experimentation. Figure 2 shows the complete experimental setup and bone models used in this study. In the CAD environment, the three-dimensional coordinates of anatomical landmarks are obtained by measuring their positions within the default coordinate system of assembly in SOLIDWORKS. Each point, such as the FHC, KC, and epicondyles, is manually selected on the digital model to define a reference configuration for the femur and tibia. In the physical setup, an infrared tracking system is used to identify the same landmarks on the 3D-printed bones. Throughout the procedure, the camera is positioned to maintain a clear line of sight to all reflective markers, while the CS−200 calibration tool is used to establish a global frame of reference. Rigid tracking markers are securely mounted on the femur and tibia to allow continuous monitoring of their spatial positions. Real-time tracking is managed through Motive 2.0 software (OptiTrack, NaturalPoint Inc., Corvallis, OR, USA), which provides the 3D coordinates of all reflectors. When registering a landmark, the tip of a tracked probe is aligned with the target point, and its location is captured via a push-button connected to an Arduino interface. At the time of capture, both the 3D position of the probe and the marker positions of the respective probe are recorded in MATLAB R2023a (MathWorks Inc., Natick, MA, USA) with reference to the global reference frame. To remove any error introduced by motion of the bone, the marked landmarks are transformed into the LCS of the bone using the fixed marker data, using Equations (3) and (4), to ensure anatomical accuracy, regardless of physical shifts during the experiment.

(8) $$G_{T}^{L}=\left[\begin{array}{cc} G_{R}^{L} & G_{p}^{L}\\ 0_{1\times 3} & 1 \end{array}\right]$$

(9) $$a_{local}=G_{T}^{L}a_{global}$$

where $G_{T}^{L}$ denotes the homogeneous transformation matrix mapping the global to the local frame, comprising a rotation matrix $G_{R}^{L}$ and translation vector $G_{p}^{L}$. The vector $a_{local}$ represents any point expressed in LCS, while $a_{global}$ is the corresponding point in the global frame.

### 2.3. Simulation and Experimental Validation of Registration Errors

**Figure 3:**
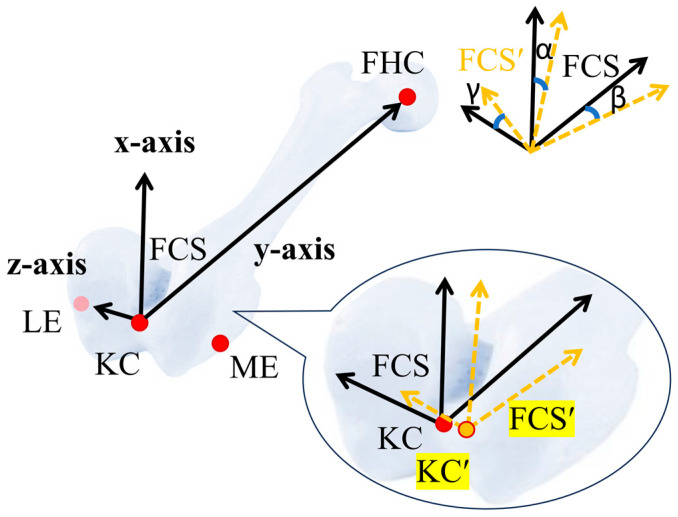
Calculation of changes in angle due to perturbation in anatomical landmark. KC is the origin for FCS; KC is the origin for FCS defined after shifting KC to a new position. α, β, γ represent cutting block angles after changing KC.

This section describes the CAD-based simulation and experimental procedures used to quantify how controlled landmark registration errors were introduced, measured, and analyzed for varus/valgus, flexion/extension, and internal/external rotation. In the CAD environment, a reference coordinate system was established using accurately marked anatomical points on CT-derived models of the femur and tibia. Controlled perturbations of ± (1, 2, and 3) mm were introduced along the global X, Y, and Z directions, corresponding to the anatomical anteroposterior, superior/inferior, and mediolateral directions, respectively. These perturbation magnitudes were selected as a bounded range of small registration offsets to evaluate directional sensitivity within a low-magnitude landmark-localization error. This choice was also informed by prior imageless TKA work in which deliberate misregistration was investigated at 2 mm intervals over a wider range [30]. This approach was used to simulate small intraoperative registration errors, in which a registered anatomical landmark may deviate from its ideal position along one or more anatomical directions, while keeping the sensitivity analysis controlled and interpretable. In the CAD analysis, perturbed landmarks were generated directly in 3D space by shifting the ideal point by prescribed offsets of ±1, ±2, and ±3 mm along the selected perturbation direction. In the experimental procedure, an arc was marked from the ideal anatomical point along the intended perturbation direction using the probe marker (Figure 2a). Because the tracking camera operates at 30 fps, approximately 30 points were recorded per second along the arc path. Each arc was traced slowly over approximately 3 s, generating about 90 recorded points along the perturbation path. These points were exported to Excel, their distances from the ideal reference point were calculated, and only those closest to the target offsets (±1, ±2, and ±3 mm) were retained for the experimental analysis. No separate repeated trials were performed for each perturbation condition. This was because the anatomical registration points had already been defined in the CAD model and were 3D printed on the bone surface, serving as fixed ground-truth references for registration, as shown in Figure 2b. For each perturbation, the coordinate system was reconstructed, and angular differences from the reference frame were calculated. The developed algorithm was then used to decompose them into clinically relevant angles: varus/valgus, flexion/extension, and internal/external rotation. To experimentally validate the CAD results, 3D-printed bone models with embedded anatomical markings (identical to those used in the CAD model) were utilized. These printed points served as ideal ground-truth references for landmark registration. The same perturbation logic applied in CAD was replicated by intentionally misregistering anatomical points during the experiment. Each perturbed point was captured, and to ensure consistency with the CAD method, transformed into a local bone coordinate system. Angular deviations were then computed relative to the ideal printed reference. By integrating virtual and physical environments, the findings enable direct comparison between simulated predictions and real-world surgical scenarios, highlighting the practical implications of registration accuracy (Figure 3).

## 3. Results

**Figure 4:**
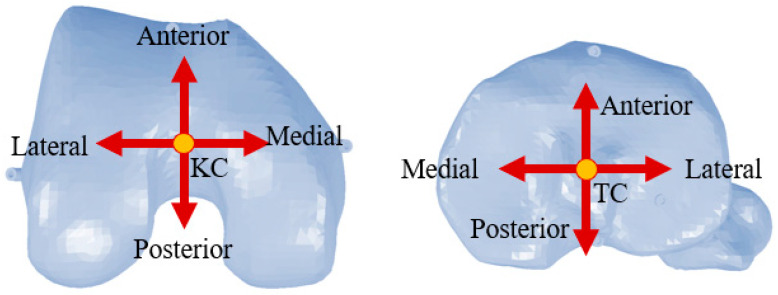
Anteroposterior and lateromedial direction where anatomical landmarks are shifted to introduce registration errors.

This section presents the angular effects of landmark shifts on femoral and tibial alignment. Femoral plots include both CAD and experimental data for varus, flexion, and rotation. Figure 4 shows the anteroposterior and lateromedial directions where the anatomical landmarks are perturbed. Overall, the most sensitive femoral responses were observed for anteroposterior perturbation of the KC in flexion/extension, mediolateral perturbation of the KC in varus/valgus, and anteroposterior perturbation of the epicondylar landmarks in internal/external rotation. On the tibial side, the strongest effects were observed for anteroposterior perturbation of the TC in slope, mediolateral perturbation of the TC in varus/valgus, and mediolateral perturbation of APX1 and APX2 in internal/external rotation.

### 3.1. Femoral Landmark Deviations

**Figure 5:**
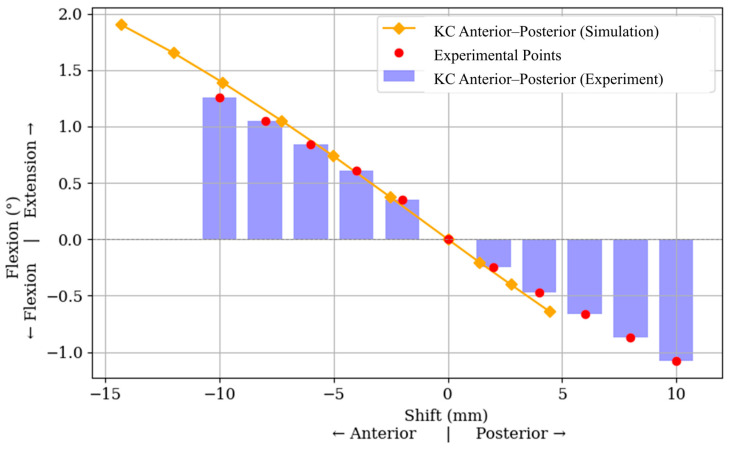
Effect of anteroposterior shift in the knee center (KC) on femoral flexion/extension. Negative KC displacement denotes anterior shift, and positive KC displacement denotes posterior shift. Positive angular values denote extension, whereas negative values denote flexion.

The anteroposterior displacement of the KC was found to have a measurable influence on knee flexion and extension. In Figure 5, negative KC displacement corresponds to anterior shift and positive displacement corresponds to posterior shift; positive angular values denote extension and negative values denote flexion. Anterior KC shifts were associated with extension, whereas posterior shifts were associated with flexion. This directional response is observed in both the simulation and experimental data across the tested displacement range. The simulation predicts a peak extension of approximately 1.9° at −14 mm, while experimental values reach 1.26° at −10 mm. In contrast, mediolateral shifts in KC and anteroposterior shifts in the femoral landmarks (i.e., ME and LE) show no measurable effect on flexion/extension, with angular deviations remaining negligible. No measurable varus/valgus change was observed for the other tested femoral landmarks in this analysis.

**Figure 6:**
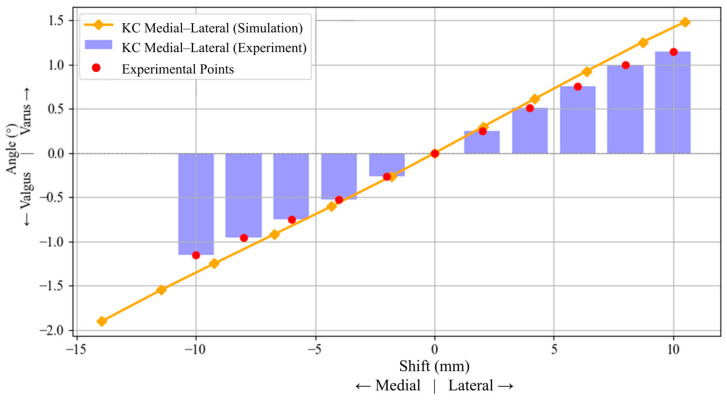
Effect of mediolateral displacement of the knee center (KC) on femoral varus/valgus alignment. Negative displacement denotes medial shift, and positive displacement denotes lateral shift. Positive angular values denote varus, whereas negative values denote valgus.

Figure 6 illustrates the influence of mediolateral shifts in the KC on varus/valgus alignment of the femur. A similar trend is observed in both simulation and experimental results: lateral shifts in the KC lead to an increase in varus alignment, whereas medial shifts result in valgus alignment. The simulation data show a nearly linear response, with varus angles increasing up to approximately 1.5° for a +10 mm lateral shift, and decreasing to −1.9° for a −14 mm medial shift. Experimental data show the same overall trend with a slightly lower magnitude, peaking at around ±1.15° for ±10 mm of lateral and medial shifts.

**Figure 7:**
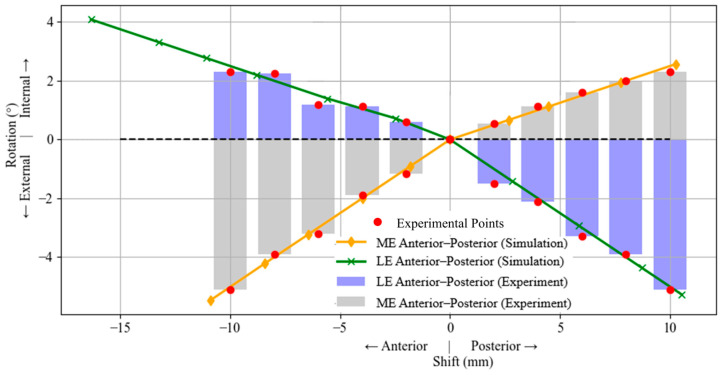
Effect of anteroposterior displacement of the medial epicondyle (ME) and lateral epicondyle (LE) on femoral internal/external rotation. Negative displacement denotes anterior shift, and positive displacement denotes posterior shift. Positive angular values indicate internal rotation, whereas negative values indicate external rotation.

Figure 7 illustrates the variation in femoral internal/external rotation due to anteroposterior shifts in the LE and ME. A similar trend is observed in both simulation and experimental data: anterior shifts lead to external rotation, while posterior shifts induce internal rotation. Experimental data for both landmarks followed the same overall trend as the simulation data. Other femoral landmarks exhibit negligible rotational influence, as shown by the near-zero values in the plot.

### 3.2. Tibial Landmark Deviations

**Figure 8:**
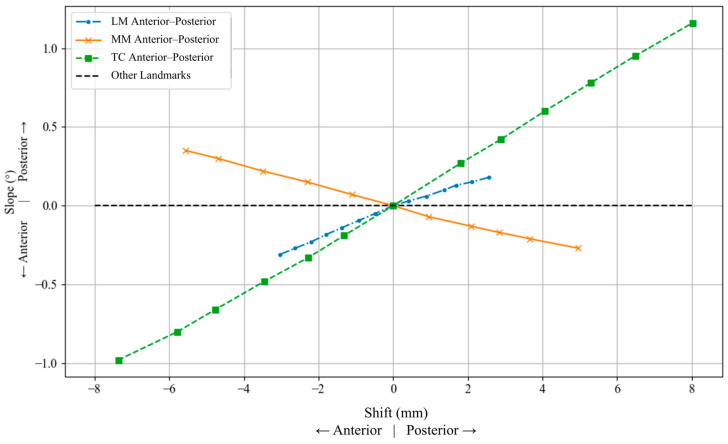
Effect of anteroposterior shifts in the lateral malleolus (LM), medial malleolus (MM), and tibial center (TC) on tibial slope (flexion/extension). Negative displacement denotes anterior shift and positive displacement denotes posterior shift, while positive and negative angular values indicate posterior and anterior slope, respectively.

The TC showed the largest change, showing a nearly linear increase in posterior slope with posterior shifts and a corresponding anterior slope with anterior shifts. The LM and MM also contributed to slope changes, but with lower sensitivity compared to TC. Figure 8 presents the effect of anteroposterior shifts in tibial landmarks on the posterior slope (flexion/extension) of the tibial plateau.

**Figure 9:**
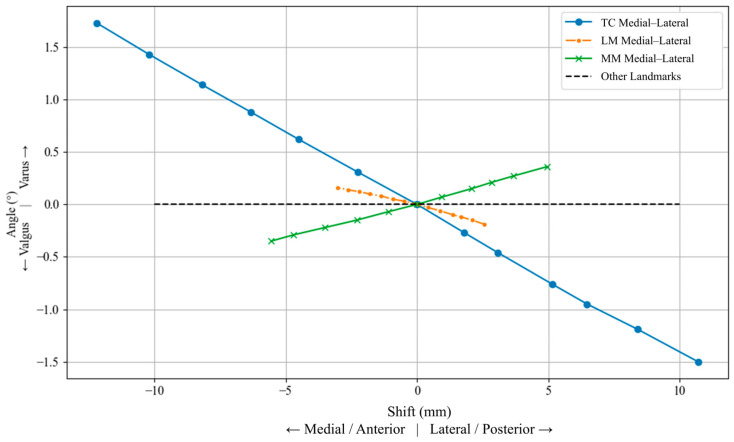
Effect of mediolateral displacement of the tibial center (TC), and anteroposterior displacement of the lateral malleolus (LM) and medial malleolus (MM), on tibial varus/valgus alignment. Negative and positive displacements denote opposite directions along the perturbation axis indicated in the legend. Positive angular values indicate varus, whereas negative values indicate valgus.

Mediolateral displacements of the TC produced the most pronounced angular variation, ranging from approximately +1.73° varus during lateral shifts, to 1.5° valgus during medial shifts. In contrast, anteroposterior perturbations of the LM and MM led to significantly smaller varus/valgus deviations. The LM caused a maximum shift of ±0.19°, while MM shifts induced deviations up to ±0.36°, both exhibiting symmetric trends about the neutral position. The LM and MM produced smaller varus/valgus deviations than the TC. Figure 9 presents the CAD-based analysis of tibial landmark shifts on varus/valgus alignment.

**Figure 10:**
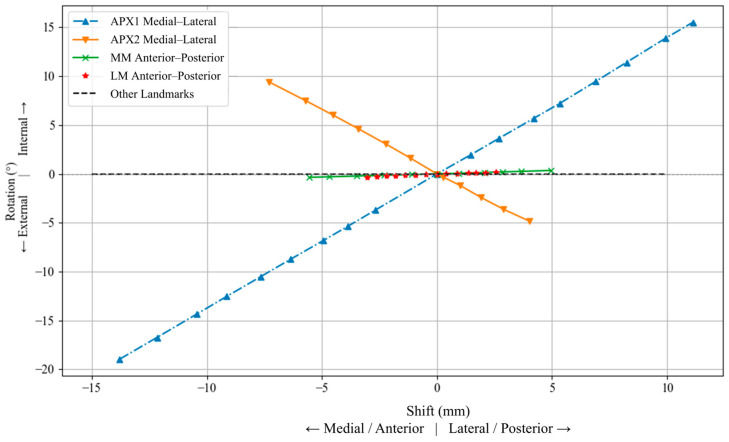
Effect of mediolateral displacement of APX1 and APX2, and anteroposterior displacement of the medial malleolus (MM) and lateral malleolus (LM), on tibial internal/external rotation.

Figure 10 demonstrates the effect of tibial landmark shifts on internal/external rotation. APX1 shifts led to a symmetric and substantial range of motion, reaching up to ~19° at ~14 mm deviation in the medial direction, and ~15° rotation at ~11 mm deviation in the lateral direction, while APX2 exhibited a slightly lower range of 4° rotation at ~5 mm rotation in the medial direction, and ~7° rotation at 9 mm of deviation in the lateral direction. In contrast, anteroposterior shifts in the MM and LM caused relatively smaller rotational changes; maximum deviations of approximately 0.35° at 5.5 mm of deviation in the anterior direction, and 0.18° at ~2.5 mm deviation in the anterior direction, respectively. The shifts in the TC and superior/inferior displacements of MM and LM showed negligible impact on rotation, consistently remaining close to zero. The largest rotational deviations were observed for mediolateral perturbations of APX1 and APX2, whereas anteroposterior perturbations of MM and LM produced comparatively small changes. Overall, the results demonstrated that the effect of registration error varied by both landmark and perturbation direction across the femoral and tibial analyses. Further interpretation of these findings is provided in Section 4.

## 4. Discussion

Both simulation and experimental data were used to systematically analyze the biomechanical influence of anatomical landmark shifts. Overall, the same directional trends were observed in both the simulation and experimental data, although the experimental magnitudes were generally lower. The lower angular deviations observed in experiments are attributed to the lack of plane-constrained point registration. In simulations, all anatomical landmarks were projected onto reference planes, defined using the anatomical point to be evaluated, ensuring pure directional shifts and maximal angular response. However, in experimental conditions, points were marked manually without reference planes, leading to off-plane deviations that blended directional components, thus dampening the observed angular effects. In addition, tracking-system accuracy, probe-tip localization uncertainty, 3D-print tolerance, and manual registration variability may also have contributed to the smaller experimental deviations. Across both segments, a common pattern was observed: shifts in the superior/inferior direction had minimal or no angular influence. This is because the anteroposterior and lateromedial anatomical points were projected onto a perpendicular plane during the coordinate system, effectively eliminating superior/inferior error components. This geometrical constraint, also visualized in Figure 1, helps isolate the true angular contribution of AP and ML deviations, and explains the flat response observed in superior/inferior shift plots. This analysis indicates that rotational and angular deviations are particularly sensitive to mediolateral and anteroposterior registration errors, especially at peripheral landmarks.

The overall trend observed in the present study is consistent with that reported in the literature, where registration error at the femoral epicondyles has been shown to produce the greatest effect on femoral rotational alignment, while other bony landmarks are comparatively more tolerant to misregistration [30]. A similar pattern was observed here, where anteroposterior perturbations of the femoral epicondyles produced the strongest internal/external rotational response, whereas tibial center perturbations mainly influenced coronal and sagittal alignment. The present study extends these earlier observations by quantifying the directional sensitivity of both femoral and tibial landmarks and by comparing CAD-based predictions with experimental validation. The reported °/mm sensitivities should be interpreted as local trends within the small perturbation range studied here, rather than as globally constant linear relationships. At larger displacements, some responses showed mild asymmetry or deviation from strict linearity. The proposed CAD-based and experimental framework was used to identify how directional registration errors at individual femoral and tibial landmarks affect cutting-plane orientation in imageless TKA. In particular, superior/inferior perturbations at most landmarks produced minimal angular deviation, whereas internal/external rotation showed the greatest sensitivity to errors affecting the lateromedial transverse-axis landmarks, thereby supporting the study hypothesis and showing a trend consistent with that reported in the literature. The reported sensitivities indicate that small registration errors at high-sensitivity landmarks may produce alignment deviations that approach the commonly accepted clinical threshold of approximately 3° [20]. Accordingly, anteroposterior and mediolateral registration accuracy should be prioritized for the KC, femoral epicondyles, tibial center, and distal AP landmarks, whereas superior/inferior perturbations at most landmarks appear less critical. From a practical standpoint, these findings suggest that greater care should be taken when registering these high-sensitivity landmarks, as small localization errors at these sites may disproportionately affect cutting-plane orientation in imageless TKA.

A limitation of this study is that the CAD analysis and experimental validation were based on a single CT-derived model and its corresponding 3D-printed specimen set, which limits generalizability. Patient-specific differences in anatomy and osteoarthritic severity may affect landmark identification and, consequently, the magnitude of error propagation and alignment sensitivity. The experimental setup was controlled and did not fully reproduce intraoperative conditions such as soft-tissue interference, limited visibility, occlusion, and mixed-direction registration errors, which may affect the direct applicability of the measured sensitivities to real TKA. Future studies should include multiple specimens with different anatomical characteristics to further validate the robustness of the proposed framework.

## 5. Conclusions

This study quantified how anatomical landmark registration errors propagate to cutting-plane orientation in imageless total knee arthroplasty using a CAD-based framework with experimental validation. The findings showed that registration-error effects are both landmark-specific and direction-dependent, with the greatest sensitivity observed in coronal and rotational alignment at selected femoral and tibial landmarks, while superior/inferior perturbations had minimal influence in most cases. These results provide practical guidance for prioritizing high-sensitivity landmarks during registration and may support refinement of imageless TKA workflows. These findings may help refine imageless TKA workflows by identifying the landmarks and perturbation directions that require the greatest registration accuracy. Future work should extend the framework to multiple anatomies and osteoarthritic severities and evaluate performance under more realistic intraoperative conditions.

## Data Availability

The original contributions presented in the study are included in the article material, further inquiries can be directed to the corresponding author.
